# Exploration of Barriers and Facilitators for Deprescribing Opioids and Benzodiazepines to Reduce Older Adult Falls

**DOI:** 10.1093/geroni/igaa057.3332

**Published:** 2020-12-16

**Authors:** Ellen Roberts, Cristine Henage, Lori Armistead, Tamera Hughes, Joshua Niznik, Courtney Schlusser, Jan Busby-Whitehead, Stefanie Ferreri

**Affiliations:** 1 ^1.^ University of North Carolina School of Medicine, Chapel Hill, North Carolina, United States; 2 ^2.^ University of North Carolina Eshelman School of Pharmacy, Chapel Hill, North Carolina, United States; 3 ^3.^ University of North Carolina Gillings School of Global Public Health, Chapel Hill, North Carolina, United States

## Abstract

As part of a randomized control trial for deprescribing opioids and benzodiazepines (BZD) to reduce falls (funded by Centers for Disease Control), we conducted a virtual focus group and surveys to evaluate opioid and BZD prescribing practices among healthcare providers in four primary care clinics in North Carolina. Survey and focus group questions measured providers’ confidence in their abilities to weigh benefits and harms of opioids and/or BZDs in older adults; determine alternative interventions; create a safe dosing plan; and incorporate patient preferences. A validated pre-intervention survey, adapted from a survey by the Canadian Deprescribing Network, was administered to providers in control and intervention clinics (n=29). Providers expressed high confidence in their abilities to weigh risks and benefits of deprescribing opioids and BZDs, but low confidence in deprescribing under impeding circumstances (e.g. when not the original prescriber or when there is no evidence to inform them). Results were similar across opioids and BZDs. A focus group was conducted among seven providers from the two intervention clinics. Barriers to deprescribing identified included patient resistance, lack of knowledge of deprescribing best practices, and lack of time to discuss deprescribing during regular clinic visits. Providers also expressed concerns about deprescribing medications initiated by or managed by other prescribers. Key facilitators of deprescribing included patient trust in physician, patients being agreeable to reduce medications, and use of gradual tapering rather than abrupt discontinuation. Barriers and facilitators were subsequently used to optimize training and provider resources for the deprescribing intervention, which is currently being implemented.

